# Megace Mystery: A Case of Central Adrenal Insufficiency

**DOI:** 10.1155/2015/147265

**Published:** 2015-12-06

**Authors:** Kunal Mehta, Irene Weiss, Michael D. Goldberg

**Affiliations:** Westchester Medical Center, Department of Medicine, Division of Endocrinology, Taylor Care Pavilion, Room D342, 100 Woods Road, Valhalla, NY 10595, USA

## Abstract

Megestrol acetate (MA) is a synthetic progestin with both antineoplastic and orexigenic properties. In addition to its effects on the progesterone receptor, MA also binds the glucocorticoid receptor. Some patients receiving MA therapy have been reported to develop clinical features of glucocorticoid excess, while others have experienced the clinical syndrome of cortisol deficiency—either following withdrawal of MA therapy or during active treatment. We describe a patient who presented with clinical and biochemical features of central adrenal insufficiency. Pituitary function was otherwise essentially normal, and the etiology of the isolated ACTH suppression was initially unclear. The use of an exogenous glucocorticoid was suspected but was initially denied by the patient; ultimately, the culprit medication was uncovered when a synthetic steroid screen revealed the presence of MA. The patient's symptoms improved after she was switched to hydrocortisone. Clinicians should be aware of the potential effects of MA on the hypothalamic-pituitary-adrenal (HPA) axis.

## 1. Introduction

When evaluating a patient who presents with central adrenal insufficiency, it is important to assess recent exposure to exogenous glucocorticoids, as this is the most common etiology of a suppressed hypothalamic-pituitary-adrenal (HPA) axis. In addition to systemic oral glucocorticoids, one must screen for the use of injectable and topical agents and for antifungal therapies (e.g., ketoconazole), opiates, and other drugs that may impair HPA axis function [1]. Megestrol acetate (MA) is a synthetic progestin approved in the United States for the palliative treatment of advanced breast and endometrial cancers and for the treatment of anorexia or weight loss in patients with AIDS. In addition to being a potent activator of the progesterone receptor, MA activates the glucocorticoid receptor, with a binding affinity almost twice that of cortisol. Patients receiving MA therapy are therefore susceptible to developing clinical dysfunction of the hypothalamic-pituitary-adrenal axis, similar to that seen with exogenous glucocorticoids.

## 2. Case Presentation

A 60-year-old female of Haitian descent presented to the emergency department with chief complaints of progressive nausea and constipation for two months. Review of systems was positive for poor appetite, fatigue, weakness, episodes of dizziness, and a recent 20-pound weight loss. The patient's medical history was significant for hypertension, type 2 diabetes, and overactive bladder, and she had undergone a total hysterectomy. Her home medications included lisinopril, metformin, oxybutynin, aspirin, and omeprazole. She denied the use of alcohol, tobacco, or illicit drugs. There were no known drug allergies. The patient was afebrile, with a blood pressure of 118/58 mm Hg. She appeared cachectic, her mucous membranes were dry, and there was poor skin turgor. She was fully oriented and in no acute distress. Cardiac, pulmonary, abdominal, and musculoskeletal examinations were unremarkable, and there were no gross neurologic deficits. Abnormalities on the blood chemistry panel included a low sodium of 130 mEq/L (normal range, 136–145), low carbon dioxide content of 10 mEq/L (normal range, 22–30), elevated creatinine of 1.8 mg/dL (normal range, 0.6–1.1), and low phosphorus of 1.4 mg/dL (normal range, 2.3–4.7). Abdominal X-ray revealed a large amount of stool in the colon, without obstruction or free air. Initial management in the emergency department included intravenous hydration, antiemetics, laxatives, and stool softeners, and the patient was admitted to the hospital for further evaluation.

Among the diagnoses considered by the admitting team was adrenal insufficiency, and a serum cortisol level was drawn at 7:00 am the next morning. When the result returned at 0.6 mcg/dL, the endocrinology service was consulted. On further questioning, the patient denied having been prescribed oral or injectable steroids or having used over-the-counter supplements of any kind. Fluticasone nasal spray had been prescribed, but it had been used only on rare occasions, and not since several months prior to admission. The patient's last menstrual period had been at age 53. She denied galactorrhea, headaches, vision changes, and frequent urination. There was no family history of pituitary or adrenal gland disease. The patient did not have phenotypic features of Cushing's syndrome or acromegaly, and there was no hyperpigmentation of the palmar creases or buccal mucosa. Blood was drawn for a repeat cortisol level, adrenocorticotropic hormone (ACTH) level, and additional pituitary hormones, after which oral hydrocortisone was started at a dose of 20 mg twice daily.

Within 24 hours, the patients' symptoms had improved and she appeared much more energetic; hydrocortisone was continued. The endocrine laboratory results are shown in Table 1. A magnetic resonance imaging (MRI) scan with and without gadolinium revealed a slightly asymmetric contour of the pituitary that was felt most likely to reflect a normal anatomic variant. On hospital day 10, a urine specimen was sent for a synthetic glucocorticoid screen. The patient was discharged home on hospital day 11 with a hydrocortisone regimen of 20 mg in the morning and 10 mg in the afternoon, potassium citrate to manage the newly diagnosed proximal renal tubular acidosis (RTA), stool softeners, and laxatives, in addition to her prior maintenance medications. Outpatient follow-up in the endocrinology clinic was planned.

The results of the urine synthetic glucocorticoid screen became available shortly after discharge. Megestrol acetate (MA) was detected, and the remainder of the screen was negative. The results were discussed with the patient over the telephone, and she denied ever having been prescribed MA. The patient's internist confirmed that this medication had not been prescribed in her office. Several days later, the patient revealed that she had HIV infection, a fact that she had previously withheld from her family, her internist, and the treating physicians in the hospital. She had been under the care of an infectious disease specialist, and in addition to being prescribed highly active antiretroviral therapy, she had been started within the past three months on MA 800 mg daily as an appetite stimulant. MA was discontinued and she is being followed in the endocrinology clinic, with the plan to gradually taper her off the glucocorticoid replacement therapy. She is also being continued on potassium citrate; her proximal RTA was felt to be related to her antiretroviral therapy.

## 3. Discussion

This patient presented to the hospital with symptomatic central adrenal insufficiency, as manifested by fatigue, dizziness, and anorexia in the context of very low serum cortisol and plasma ACTH levels (see Table 1). Given the frank degree of suppression of these hormones, we did not feel it was necessary to perform a cosyntropin stimulation test to confirm the diagnosis. There was no biochemical evidence for additional anterior pituitary hormone deficiencies, nor was there hyperprolactinemia, though the gonadotropin levels were somewhat lower than expected for the postmenopausal state. The very slight elevation in IGF-1, in the absence of any clinical features of acromegaly, was felt most likely to be a nonsignificant result. Therefore, our clinical impression was that the patient had isolated ACTH suppression; the etiology, however, was not readily apparent.

The differential diagnosis of isolated ACTH deficiency includes genetic defects such as mutations in the proopiomelanocortin (POMC) gene [2] and in the gene for TPIT, a T-box transcription factor involved in the differentiation of corticotrophs [3]. However, these are rare conditions that typically present in infancy or childhood. Therefore, the use of exogenous glucocorticoids was strongly suspected to be the cause of our patient's central adrenal insufficiency. However, other than the infrequent use of nasal fluticasone several months prior to admission, she denied having taken any steroid preparations. Ultimately, the culprit drug, megestrol acetate, was detected using a urine synthetic glucocorticoid screen. Even after being confronted with this laboratory finding, it took some time for the patient to admit to having been prescribed megestrol acetate by an infectious disease specialist, as she had not yet revealed her HIV-positive status to her family or her primary care physician.

Megestrol acetate (MA) is an orally active, synthetic 17-hydroxyprogesterone derivative. It is approved by the United States Food and Drug Administration for the palliative treatment of advanced breast and endometrial cancers and for the treatment of anorexia, cachexia, or unexplained weight loss in patients with AIDS. Though the exact mechanism of its antineoplastic action remains unclear, it is postulated that progesterone receptor binding leads to feedback inhibition of gonadotropin releasing hormone and gonadotropins, which results in a reduction in circulating sex steroid hormones and downregulation of androgen and estrogen receptors [4]. Of note, this may explain the lower-than-expected gonadotropin levels that were seen in our patient. The mechanism by which MA stimulates appetite also has yet to be fully elucidated [5].

Many naturally occurring and synthetic steroid hormones bind to multiple steroid hormone receptors, and MA is no exception. In 1983, Kontula et al. demonstrated that MA displayed a considerable binding affinity to the glucocorticoid receptor (GR), almost twice that of the natural ligand cortisol [6]. Once MA was introduced into clinical practice, reports appeared in the literature describing biochemical evidence of endogenous HPA axis suppression in patients taking this medication [7–9]. In addition, reports emerged describing weight gain, new onset diabetes, worsening hyperglycemia in patients with preexisting diabetes, or the development of Cushingoid features, in patients receiving MA for advanced hormone-responsive cancers [10–12] and for AIDS cachexia [13–15]. These features of glucocorticoid excess typically resolved after discontinuation of the MA therapy. A survey of adverse drug event reports submitted to the US Food and Drug Administration between 1984 and 1996 revealed a total of 12 cases of new onset hyperglycemia, 12 cases of worsening hyperglycemia in patients with diabetes, and 5 cases of frank Cushing's syndrome in patients receiving MA [16].

In patients who have been treated chronically with exogenous glucocorticoids, abrupt withdrawal of these agents may result in the clinical syndrome of adrenal insufficiency. Given the ability of MA to bind the glucocorticoid receptor and produce symptoms and signs of glucocorticoid excess, while suppressing endogenous cortisol secretion, one expects that patients withdrawn from MA therapy may also be at risk for a steroid withdrawal syndrome. Case reports have described such an occurrence, with patients experiencing prompt clinical improvement after the initiation of oral hydrocortisone treatment [14, 17]. Our patient, however, developed clinically significant central adrenal insufficiency while* actively* receiving treatment with MA—not following its withdrawal. This has been reported to occur in patients receiving MA therapy for hormone-responsive cancers [18] and for cachexia syndromes [19, 20], as well as in a population of acutely ill hospitalized patients [21]. The aforementioned review of adverse events submitted to the FDA uncovered a total of 16 cases of clinically apparent adrenal insufficiency between 1984 and 1996, with some occurring during active treatment with MA and others developing after its discontinuation [16]. Daily doses of MA in these reports ranged from 60 to 1,600 mg, and durations of therapy ranged from days to months.

The mechanism by which the active use of an agent with glucocorticoid-like activity, not its withdrawal, may cause clinical adrenal insufficiency has yet to be fully elucidated. Mann et al. outlined a number of possible explanations, including unreported discontinuation of MA, the presence of intercurrent acute stress or illness in the context of an already suppressed HPA axis, a dual agonist-antagonist action of MA (binding to the GR as a weak agonist, but also antagonistically blocking the binding of more potent endogenous glucocorticoids to the GR), and an inherently greater potential for MA to suppress the HPA axis than to induce glucocorticoid-like clinical effects [16]. Leinung et al. proposed several factors that may contribute to the clinical variability in individual responses to MA, including the apparent combined central agonism/peripheral antagonism observed clinically in the subset of patients with HPA axis suppression and symptomatic glucocorticoid deficiency. These included variations in pharmacokinetic properties of MA across individuals, the effects of progestational actions of MA on the mineralocorticoid receptor, and differences in the interactions of the MA-GR complex with DNA and transcription modulators across different tissues [22].

To our knowledge, this is the first report of MA-induced central adrenal insufficiency in which the patient's use of MA was unknown at the time of initial presentation. This case highlights, therefore, the clinical utility of the urine synthetic glucocorticoid screen when the etiology of isolated ACTH suppression is not readily apparent. This panel, which is performed at the Mayo Medical Laboratories, utilizes a high-performance liquid chromatography system and tandem mass spectrometry (LC-MS/MS) to detect the current or recent use of fourteen synthetic steroids [23]. While much of the literature on synthetic glucocorticoid analyses has focused on veterinary applications [24], or screening for “doping” in athletes [25], the value of such testing has also been demonstrated in the contexts of factitious Cushing's syndrome [26] and cases of clinical adrenal dysfunction due to intra-articular and epidural glucocorticoid administration [27].

In summary, clinicians are well advised to consider the potential for megestrol acetate therapy to cause adrenal dysfunction. Clinical manifestations of adrenal insufficiency may occur after abrupt withdrawal of MA, or even during active treatment, as was seen in our patient. Additionally, when the cause of central adrenal insufficiency is difficult to ascertain, the use of a synthetic glucocorticoid screen can be invaluable in uncovering the occult use of traditional oral, parenteral, or topical steroids, as well as megestrol acetate: a progestin with important glucocorticoid activity.

## Figures and Tables

**Table 1:** Endocrine lab results.

	Hospital day 1, 7:00 am	Hospital day 1, 5:30 pm	Hospital day 5	Normal range
Cortisol	0.6 mcg/dL	1.0 mcg/dL		6.2–19.4
Adrenocorticotropic hormone (ACTH)		<5 pg/mL		10–60
Thyroid stimulating hormone (TSH)		1.05 mIU/L		0.35–4.70
Free thyroxine		0.8 ng/dL		0.7–1.9
Luteinizing hormone (LH)		12.0 mIU/mL		
Follicle stimulating hormone (FSH)		28.6 mIU/mL		
Prolactin		16.0 ng/mL		1.2–29.9
Insulin-like growth factor-1 (IGF-1)			178 ng/mL	45–173
